# Use of a self-completed life history calendar in relation to data completeness and accuracy

**DOI:** 10.1186/s12874-026-02777-5

**Published:** 2026-02-05

**Authors:** Jennifer Yu, Prevost Jantchou, Rui Ning Gong, Belinda Nicolau, Sreenath Madathil, Miceline Mesidor, Marie-Claude Rousseau

**Affiliations:** 1 Epidemiology and Biostatistics Unit, Centre Armand-Frappier Santé Biotechnologie, Institut national de la recherche scientifique (INRS), 531 Boul. des Prairies, Laval, QC H7V 1B7 Canada; 2 Research Centre, CHU Sainte-Justine, 3175 ch. de la Côte-Sainte-Catherine, Montréal, QC H3T 1C5 Canada; 3 Department of Pediatrics, Division of Gastroenterology, Hepatology, and Nutrition, CHU Sainte-Justine, 3175 Chemin de la Côte-Sainte-Catherine, Montréal, QC H3T 1C5 Canada; 4 Faculty of Dental Medicine and Oral Health Sciences, McGill University, 2001 McGill College, Montréal, QC H3A 1G1 Canada; 5 Faculty of Pharmacy, Université de Montréal, Pavillon Jean-Coutu, 2940, chemin de Polytechnique, Montréal, QC H3T 1J4 Canada; 6 School of Public Health, Université de Montréal, 7101 av. du Parc, Montréal, QC H3N 1X9 Canada; 7 Innovation Hub, Centre de recherche du Centre hospitalier de l’Université de Montréal (CRCHUM), 900 Saint-Denis, Montréal, QC H2X 0A9 Canada

**Keywords:** Life history calendar, Data completeness, Agreement, Memory aid, Life course epidemiology, Retrospective data collection

## Abstract

**Background:**

A life history calendar allows mapping personal events to improve recall of past events in retrospective data collection. We estimated whether the use of a life history calendar was related to data completeness and accuracy.

**Methods:**

Participants in a case-control study on inflammatory bowel disease in Quebec, Canada in 2021, were invited to complete a preparatory life history calendar and encouraged to consult it during data collection. For data completeness, associations between life history calendar preparation/consultation frequency and number of missing values were estimated using negative binomial regression (Sample Mean Ratio, SMR) with inverse-probability-weighting to balance participants’ characteristics across life history calendar preparation/consultation groups. One hundred and thirty variables were considered. For accuracy, parents’ age at participants’ birth and number of older siblings, the only variables available from both the questionnaire and Birth Registry, were compared for agreement according to life history calendar preparation.

**Results:**

Of 2727 participants, 48% prepared the life history calendar. During data collection, 27%, 48%, and 25% consulted it never, sometimes, often/always, respectively. The overall proportion of missing values was low (0.7%). Life history calendar preparation (vs. not) was associated with a 22% decrease in number of missing values (SMR = 0.78; 95% CI: 0.64–0.96). There were fewer missing values with greater consultation frequency, with 26% (SMR = 0.74; 95% CI: 0.56–0.98), 31% (SMR = 0.69; 95% CI: 0.55–0.86) and 59% (SMR = 0.41; 95% CI: 0.29–0.56) fewer missing values among those who never, sometimes, often/always consulted it, respectively, vs. not having completed a life history calendar. Life history calendar preparation was associated with slightly better agreement between self-reported and registry data for mother’s (5% higher) and father’s (4% higher) age at participant’s birth, but not number of older siblings (1% lower).

**Conclusions:**

Life history calendar preparation/consultation were associated with higher data completeness. However, the assessment of data accuracy was limited due to the small number of available variables.

**Supplementary Information:**

The online version contains supplementary material available at 10.1186/s12874-026-02777-5.

## Background

Life course research on chronic diseases aims to study the long-term effects of exposures throughout an individual’s life on health outcomes later in life [1]. These exposures are often documented retrospectively, through surveys or questionnaires which are prone to recall bias, misclassification, and missing data [2]. The life history calendar (LHC) is a tool designed to overcome these issues [3]. It has also been called an “event history calendar” or a “life grid”, and is designed as a grid with the timeline on one axis and different life domains on the other axis. The timeline may span from a few months to a lifetime, with units of time such as days, months, or years. Life domains typically include the residential, educational, and occupational histories, major life events, and sometimes public landmark events [2]. The LHC stimulates memories sequentially within the same sphere of life and in parallel between spheres, allowing a general overview of the subject’s life circumstances, in order to recall past events [4].

The LHC has been used in research on mental health [5], migration [6], sexual health and relationships [7], and occupational exposures [8]. It may replace a conventional questionnaire where the lists of questions are organized by themes [6–9]. It may also be used as a memory aid to a conventional questionnaire [10–15], where “flashbulb events” could prompt the memory of related events, by an anchoring mechanism [16, 17]. While a LHC may require guidance due to its complexity [18], past studies have shown that it can be self-completed effectively [14, 19, 20].

The LHC enhances data quality and consistency at multiple data collection points and aligns well with information from other data sources [21]. However, only a few studies have investigated its impact on data completeness. A small study reported no association [20], another reported that LHC use increased data completeness only for distant events [19], while a third study found a positive association overall [11]. These studies were methodologically different: the sample size (range from 138 to 2493 participants), the purpose of the LHC (as the primary study questionnaire or as a memory aid), and the administration method (self-administered or interviewer-administered). A common limitation in these studies was that they used the number of events to determine data completeness, equating a higher number of reported events to better data completeness. However, different individuals may have experienced different numbers of events, so this is not a direct measure of data completeness. To address this issue, we compared the number of missing values from an equal number of variables from the study questionnaire of a case-control study of inflammatory bowel diseases, between participants with or without a self-administered LHC. Additionally, we assessed the agreement between self-reported information and administrative records in relation to the use of LHC.

## Methods

### Study design

In 2021, we conducted a case-control study on inflammatory bowel diseases (Crohn’s disease and ulcerative colitis) and lifetime exposures likely to influence the intestinal microbiota. This case-control study was part of the expanded Quebec Birth Cohort on Immunity and Health (CO·MMUNITY), including 400,611 persons born in the province of Quebec between 1970 and 1974 [22, 23]. We identified Crohn’s disease and ulcerative colitis cases using validated algorithms based on administrative health data [24, 25], and randomly sampled controls among cohort subjects without inflammatory bowel disease. Our final sample included 1212 Crohn’s disease cases, 570 ulcerative colitis cases and 946 controls, resulting in participation rates of 52%, 55%, and 47%, respectively. The data collection was carried out through online surveys or phone interviews with participants aged 46 to 51 years old. The data spanned from birth until 2014, the last year for which administrative health data was available for inflammatory bowel diseases identification, and thus the last year for which we asked information from the participants.

We obtained the necessary authorizations for data access from the Quebec governmental authorities (Commission d’accès à l’information) and the Research Ethics Committees of the Institut national de la recherche scientifique, Institut de la statistique du Québec, Centre hospitalier universitaire Sainte-Justine and Régie de l’assurance maladie du Québec. All participants have provided informed consent (online or orally) for their participation in the study and for the linkage of their collected data with administrative data.

### Recruitment

During recruitment by the Institut de la statistique du Québec, potential participants received a letter introducing the study, a LHC, and a list of items to help them prepare for questions that may be more difficult to remember (birth circumstances, feeding in infancy, kindergarten attendance, parents’ socio-demographic characteristics, and antibiotic use throughout life).

### Construction of LHC

**Fig. 1:**
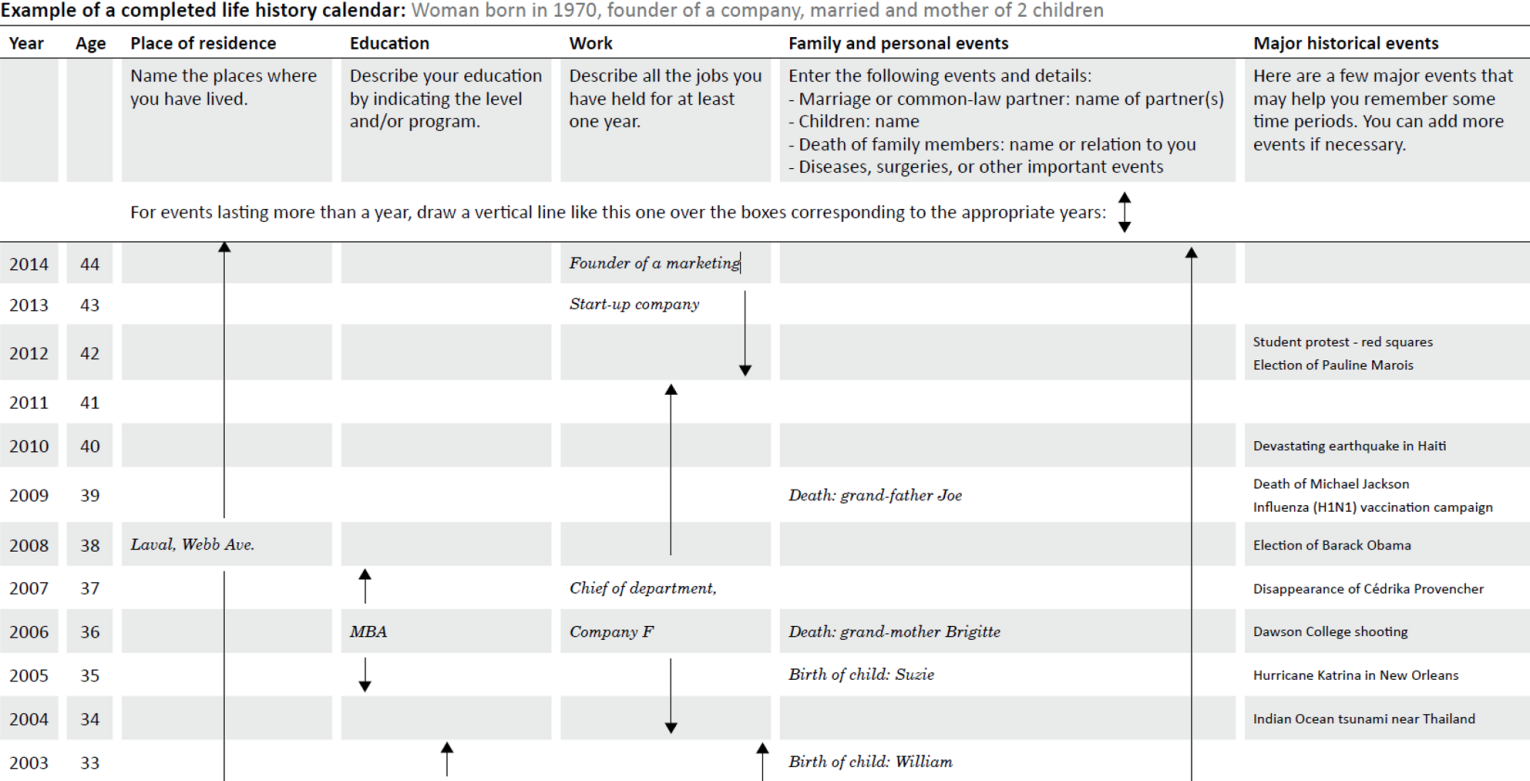
Excerpt of a completed life history calendar for a fictional participant

Inspired from a previous LHC [20], we designed a self-administered, two-page paper memory aid (Fig. 1) to be completed prior to the main conventional structured questionnaire. It had the form of a grid where each row represented an age and the corresponding calendar year. The columns included the residential, schooling and occupational histories, familial and personal events, and major historical events. Instructions and a filled example were included. Prior to the recruitment of participants, a pretest was conducted on the LHC and the main questionnaire to ensure question clarity and that the major historical events in the LHC were relevant to the target population. 

### Main study questionnaire

Participants were encouraged to complete the main questionnaire online by themselves but could also complete it on the phone with a trained interviewer. At the beginning of the main questionnaire, the participants were asked whether they prepared the LHC (yes or no), and they were reminded to consult the calendar while filling out the questionnaire. At the end of the main questionnaire, participants reported how often they consulted the LHC (never, sometimes, often, always, or almost always).

Sections of the main questionnaire included family socio-demographic background, birth circumstances, infancy feeding, kindergarten attendance, body silhouettes, pet ownership, smoking, diet and physical activity, intestinal health and family history, reproductive health, hormonal treatments, antibiotic treatments, and psychosocial factors. More details on the specific questionnaire items and sources of questions are provided elsewhere [23]. There was a difference in the choice of “I don’t know” as an answer online and by telephone. In the online questionnaire, this option was left out to encourage participants to provide an answer. If a participant wanted to skip a question, they got a prompt that an answer was missing, although they could still skip it. Participants on the phone could say they didn’t know the answer, or they didn’t want to answer. The interviewers were instructed to mention to participants that their best guess would be better than not responding. Yet, participants had the option of responding “I don’t know”. 

### Statistical analyses

#### Data completeness

Data completeness was measured by the number of missing values, with a complete dataset having no missing value. A missing value was defined either as a “I don’t know” or a “No response”, both answers treated as equivalent.

We generated three groups of variables (Table S1 in Additional file 1): (1) *Main variables* (*n* = 130); (2) *Date variables* (*n* = 30); (3) *Childhood variables* (*n* = 45). The latter two groups of variables were subsets of the *Main variables*. *Main variables* included all variables from the questionnaire judged to be emotionally neutral, excluding psychosocial factors. The *Date variables* included years or ages at exposures or events. The *Childhood variables* included data from birth to childhood typically harder to recall due to time passed.

Multiple imputation by chained equations (MICE) was carried out for confounding variables with missing values: European socio-economic classification [26], derived from the longest occupation, education level, administrative data-derived residential area in 2014 (rural vs. urban), median census-based income quartile in 2011, ethnic origins, mother’s education level, material deprivation index quintile in 2014 [27], social deprivation index quintile in 2014 [27], father’s education level. These variables were not used for the count of missing values in the *Main variables*, *Date variables*, nor *Childhood variables*. Predictors used in MICE were the imputed variables listed above and the following variables with complete data: inflammatory bowel diseases status, sex, birth year and mode of data collection (online vs. by phone). Given that 12% of participants had at least one missing value, 12 imputed datasets were generated.

To model the number of missing values, negative binomial regression was used after verifying that it had the optimal BIC value compared to Poisson, to zero-inflated Poisson or to zero-inflated negative binomial models (Table S2 in Additional File 2). Results are presented as sample mean ratios (SMR) of number of missing values with 95% confidence intervals. A regression model was estimated for the association between each combination of exposure [LHC preparation (yes or no); LHC consultation frequency (not prepared, prepared but never consulted, prepared and sometimes consulted, and prepared and often/always/almost always consulted)], and outcome [number of missing values for the *Main variables*, for *Date variables*, or for *Childhood variables*], along with the mode of data collection as a confounder.

LHC preparation and consultation frequency may have been linked to participant characteristics that are also related to data completeness and accuracy, such as education level. To achieve a balance of participant characteristics across the exposure groups, propensity scores for the probability of LHC preparation/consultation were estimated by logistic regression using all the confounding variables except the mode of data collection. The propensity scores were then converted into stabilized inverse probability weights – the inverse of the probability of being in an exposure group (LHC prepared versus not prepared, or different consultation frequencies) [28]. We checked the validity of propensity scores with stabilized inverse probability weighting via standardized mean differences: a value less than 0.1 (Table 1) indicated a balanced characteristic across exposure groups. The weights were integrated in each of the negative binomial regression models.

**Table 1:** Participant socio-demographic characteristics according to the preparation of the life history calendar

	LHC prepared *n* = 1297 (%)	LHC not prepared *n* = 1430 (%)	SMD before weighting^a^	SMD after weighting^a^
Inflammatory bowel disease status				
Control	418 (32)	528 (37)	-0.10	0.01
Crohn’s disease	596 (46)	616 (43)	0.06	-0.01
Ulcerative colitis	283 (22)	286 (20)	0.05	0.004
Sex				
Men	399 (31)	609 (43)	NA	NA
Women	898 (69)	821 (57)	0.25	-0.01
Birth year				
1970	276 (21)	337 (24)	-0.06	-0.01
1971	281 (22)	279 (20)	0.05	-0.01
1972	230 (18)	274 (19)	-0.04	0.02
1973	247 (19)	262 (18)	0.02	-0.004
1974	263 (20)	278 (19)	0.02	0.01
Mode of data collection				
Phone	94 (7)	353 (25)	NA	NA
Online	1203 (93)	1077 (75)	0.49	0.48
Grandparents’ ethnic origins				
French only	946 (73)	983 (69)	0.09	-0.01
At least one French	225 (17)	240 (17)	0.02	-0.004
At least one British	39 (3)	42 (3)	0.004	0.01
Other only	80 (6)	146 (10)	-0.15	0.01
Unknown	7 (0.5)	19 (1)	-0.08	NA
Father’s education level				
Primary	547 (42)	597 (42)	0.01	0.004
Secondary	438 (34)	437 (31)	0.07	-0.02
College	58 (5)	66 (5)	-0.01	0.0004
University	225 (17)	222 (16)	0.05	0.02
Unknown	29 (2)	108 (8)	-0.25	NA
Mother’s education level				
Primary	499 (38)	561 (39)	-0.02	0.01
Secondary	526 (41)	562 (39)	0.03	-0.01
College	103 (8)	95 (7)	0.05	-0.003
University	152 (12)	127 (9)	0.09	-0.01
Unknown	17 (1)	85 (6)	-0.25	NA
Participant’s highest achieved education level				
Primary	55 (4)	124 (9)	-0.18	-0.01
Secondary	358 (28)	503 (35)	-0.16	0.01
College	342 (26)	332 (23)	0.07	-0.002
University	538 (42)	4666 (33)	0.19	-0.003
Unknown	4 (0.3)	5 (0.3)	-0.01	NA
European socio-economic classification of occupation				
Salariat	545 (42)	530 (37)	0.10	0.002
Intermediate	402 (31)	399 (28)	0.07	0.01
Working class	326 (25)	469 (33)	-0.17	-0.02
Unemployed/Not classified	24 (2)	32 (2)	-0.03	0.02
Median income quartile in 2011				
Quartile 1 (lowest income)	279 (22)	349 (24)	-0.07	0.02
Quartile 2	297 (23)	383 (27)	-0.09	-0.001
Quartile 3	329 (25)	322 (23)	0.07	-0.004
Quartile 4 (highest income)	383 (30)	363 (25)	0.09	-0.02
Unknown	9 (0.7)	13 (0.9)	-0.02	NA
Material deprivation index quintile in 2014				
Quintile 1 (lowest deprivation, most advantaged)	330 (25)	292 (20)	0.12	0.01
Quintile 2	277 (21)	307 (22)	-0.003	-0.01
Quintile 3	273 (21)	271 (19)	0.05	0.02
Quintile 4	213 (16)	273 (19)	-0.07	-0.03
Quintile 5 (highest deprivation, least advantaged)	153 (12)	224 (16)	-0.11	0.01
Unknown	51 (4)	63 (4)	-0.02	NA
Social deprivation index quintile in 2014				
Quintile 1 (lowest deprivation, most advantaged)	329 (25)	352 (25)	0.02	-0.02
Quintile 2	265 (20)	322 (23)	-0.05	0.01
Quintile 3	289 (22)	285 (20)	0.06	-0.0003
Quintile 4	211 (16)	224 (16)	0.02	-0.01
Quintile 5 (highest deprivation, least advantaged)	152 (12)	184 (13)	-0.04	0.03
Unknown	51 (4)	63 (4)	-0.02	NA
Residential area in 2014				
Rural	245 (19)	290 (20)	-0.04	0.01
Urban	1048 (81)	1131 (79)	0.04	NA
Unknown	4 (0.3)	9 (0.6)	-0.05	NA

Compilation based on data from the ©Government of Québec, Institut de la statistique du Québec, CO·MMUNITY, 2017. ©Government of Québec, Institut de la statistique du Québec, Life History Intestinal Health Study, 2021. Institut de la statistique du Québec is not responsible for compilations or interpretation of results

*LHC* Life history calendar, *SMD* Standardized mean difference – difference in percentage of effect size between groups divided by the standard deviation within group. A SMD of > 0.1 is considered to be indicative of a difference. For binary variables, there is only one SMD value because if one category is unbalanced, the other category is automatically unbalanced

^a^ Multiple imputation by chained equations conducted on missing values of variables which were used for propensity score and stabilized inverse probability of treatment weight calculations for balancing characteristics between groups. SMDs are presented for first of 12 imputed datasets. No SMD value are present for the “Unknown” category because category no longer existed after imputation

#### Agreement between data sources

Data agreement in relation to LHC preparation was investigated for the variables documented in two sources, our study questionnaire and the Quebec birth registry: father’s age and mother’s age at participant’s birth, and number of older siblings. In the main questionnaire, the first data source, we asked participants for their parent’s birth year and number of older siblings. We then converted their parents’ birth year into an age by subtracting the participant’s birth year from the parents’ birth years. In the Quebec birth registry, the second data source, we had the exact age of the parents when the participant was born, and the number of children the mother had before the participant’s birth. Since we had an exact parents’ age in the administrative data, but not in the questionnaire, a difference of one unit was tolerated for agreement. Subgroup agreement rates were calculated according to LHC preparation.

Statistical analyses were carried out using the R statistical program (R Foundation, Vienna, Austria) [29] with the packages cobalt [30], mice [31], and WeightIt [32].

## Results

### Participant characteristics

One participant did not report whether they prepared the LHC and was excluded. In total, 1297 (48%) participants prepared the LHC, and 1430 (52%) did not. Of those who prepared it, nine did not report their frequency of consultation during data collection, 352 (27%), 616 (47%), and 320 (25%) reported never, sometimes, and often, almost always, or always consulting it, respectively. Among participants who prepared the LHC, 93% completed the questionnaire online; in participants who did not prepare the LHC, 75% completed the questionnaire online. Also, among those who prepared the LHC compared to those who didn’t, there was a higher proportion of cases, women, and participants with a higher socio-economic position based on education, occupation, census-based income, and deprivation indices (Table 1). Notable differences in participant characteristics were evident when comparing respondents who completed the main questionnaire online to those who participated via phone. The latter group tended to include a higher proportion of cases, individuals living in rural areas and lower socio-economic position including lower education level, working class occupations, higher material deprivation and lower median income. 

### Data completeness

The proportion of missing values in the overall questionnaire was 0.7%. Notably, the reproductive history and medication section of the questionnaire had the highest percentage of missing values (1.6%), followed by the sociodemographic (1.4%) and childhood sections (1.4%). All other sections presented less than 1% of missing values.

Among the participants who did not prepare the LHC, 35% had at least one missing value within the *Main variables* with a mean of 1.2 (SD = 2.7) missing values per participant. In contrast, among those who did prepare the LHC, this percentage was lower at 17% with a mean of 0.51 (SD = 2.7) missing value per participant (Table 2). Similarly, the proportion of participants with missing values in the *Date* and *Childhood variables* was lower among those who prepared the LHC than those who didn’t. Likewise, the number of missing values was lower among the former than the latter. When considering consultation of the LHC, the percentages of participants with at least one missing value in the *Main variables* were 17%, 18%, and 13% for those reporting never, sometimes and often, almost always, or always consulting it, respectively.

**Table 2:** Associations between life history calendar preparation and number of missing values

	Mean *n* missing values per participant (SD)	*n* participants with at least one missing value (%)	Unadjusted and unweighted sample mean ratio (SMR, 95% CI)	Adjusted and weighted sample mean ratio (SMR, 95% CI)^a^
Main variables				
LHC not prepared (*n* = 1430)	1.2 (2.7)	503 (35)	1.00 (Reference)	1.00 (Reference)
LHC prepared (*n* = 1297)	0.51 (2.7)	226 (17)	0.42 (0.34–0.51)	0.78 (0.64–0.96)
Date variables				
LHC not prepared (*n* = 1430)	0.19 (0.61)	173 (12)	1.00 (Reference)	1.00 (Reference)
LHC prepared (*n* = 1297)	0.08 (0.41)	72 (6)	0.42 (0.31–0.57)	0.63 (0.47–0.86)
Childhood variables				
LHC not prepared (*n* = 1430)	0.64 (1.5)	350 (24)	1.00 (Reference)	1.00 (Reference)
LHC prepared (*n* = 1297)	0.22 (1.2)	128 (10)	0.33 (0.26–0.42)	0.73 (0.59–0.92)

Compilation based on data from the ©Government of Québec, Institut de la statistique du Québec, CO·MMUNITY, 2017. ©Government of Québec, Institut de la statistique du Québec, Life History Intestinal Health Study, 2021. Institut de la statistique du Québec is not responsible for compilations or interpretation of results

*CI* Confidence interval, *LHC* Life history calendar, *SD* Standard deviation, *SMR* Sample mean ratio computed from negative binomial regression analyses

^a^ Adjusted for mode of data collection and model weighted with inverse probabilities of LHC preparation based on sex, inflammatory bowel disease case status, birth year, European socio-economic classification, participant’s education level, residential area in 2014 (rural vs. urban), median income quartile in 2011, ethnic origins, mother’s education level, material deprivation index quintile in 2014, social deprivation index quintile in 2014, father’s education level

The mode of data collection revealed the most notable difference in the occurrence of missing values for the *Main variables*: 76% of phone interviews had at least one missing value (mean = 3.0 missing values, SD = 3.6), whereas 17% of online surveys had any missing values (mean = 0.47 missing values, SD = 2.3).

In the negative binomial regression analyses (Table 2), we observed that participants who prepared the LHC had an average of 22% fewer missing values in the *Main variables* compared to participants who did not prepare the LHC with a 95% confidence interval ranging from 4% to 36% (SMR = 0.78, 95% CI = 0.64–0.96). The results did not differ from those of zero-inflated negative binomial models (Table S3 in Additional File 3). For *Date* and *Childhood variables*, the disparities were even more pronounced. In comparison to those who did not, those who prepared the LHC had 37% (95% CI = 14–53%) fewer missing values in the *Date* variables and 27% (95% CI = 8–41%) fewer in the *Childhood* variables. Additionally, across all three categories, *Main*, *Date* and *Childhood variables*, there was a consistent trend: as LHC consultation frequency increased, the SMR values decreased (*p*-values for trend < 0.01, Table 3).

**Table 3:** Associations between life history calendar consultation frequency and number of missing values

	Mean *n* missing values per participant (SD)	*n* participants with at least one missing value (%)	Unadjusted and unweighted sample mean ratio (SMR, 95% CI)	Adjusted and weighted sample mean ratio (SMR, 95% CI)^a^	*P* for trend
Main variables					< 0.001
LHC not prepared (*n* = 1430)	1.2 (2.7)	503 (35)	1.00 (Reference)	1.00 (Reference)	
LHC prepared, never consulted (*n* = 352)	0.46 (1.7)	61 (17)	0.38 (0.28–0.52)	0.74 (0.56–0.98)	
LHC prepared, sometimes consulted (*n* = 616)	0.45 (1.4)	113 (18)	0.37 (0.29–0.48)	0.69 (0.55–0.86)	
LHC prepared, often or almost always or always consulted (*n* = 320)	0.35 (1.3)	43 (13)	0.29 (0.21–0.40)	0.41 (0.29–0.56)	
Date variables					0.003
LHC not prepared (*n* = 1430)	0.19 (0.61)	173 (12)	1.00 (Reference)	1.00 (Reference)	
LHC prepared, never consulted (*n* = 352)	0.07 (0.32)	20 (6)	0.36 (0.21–0.59)	0.50 (0.30–0.84)	
LHC prepared, sometimes consulted (*n* = 616)	0.08 (0.43)	32 (5)	0.40 (0.27–0.59)	0.59 (0.40–0.88)	
LHC prepared, often or almost always or always consulted (*n* = 320)	0.05 (0.29)	13 (4)	0.28 (0.15–0.48)	0.36 (0.19–0.65)	
Childhood variables					0.001
LHC not prepared (*n* = 1430)	0.64 (1.5)	350 (24)	1.00 (Reference)	1.00 (Reference)	
LHC prepared, never consulted (*n* = 352)	0.20 (0.84)	34 (10)	0.31 (0.21–0.46)	0.67 (0.48–0.94)	
LHC prepared, sometimes consulted (*n* = 616)	0.18 (0.72)	64 (10)	0.28 (0.21–0.39)	0.61 (0.46–0.80)	
LHC prepared, often or almost always or always consulted (*n* = 320)	0.18 (0.76)	27 (8)	0.29 (0.19–0.43)	0.52 (0.35–0.75)	

Compilation based on data from the ©Government of Québec, Institut de la statistique du Québec, CO·MMUNITY, 2017. ©Government of Québec, Institut de la statistique du Québec, Life History Intestinal Health Study, 2021. Institut de la statistique du Québec is not responsible for compilations or interpretation of results

*CI* Confidence interval, *LHC* Life history calendar, *SD* Standard deviation, *SMR* Sample mean ratio computed from negative binomial regression analyses

^a^ Adjusted for mode of data collection and model weighted with inverse probabilities of LHC preparation based on sex, inflammatory bowel disease case status, birth year, European socio-economic classification, participant’s education level, residential area in 2014 (rural vs. urban), median income quartile in 2011, ethnic origins, mother’s education level, material deprivation index quintile in 2014, social deprivation index quintile in 2014, father’s education level

### Agreement between data sources

A total of 124 participants were excluded due to missing values related to the father’s age at the participant’s birth in either or both data sources, 75 participants for mother’s age and 74 for the number of older siblings. There was a slightly higher level of agreement between the two data sources for the father’s and mother’s age at participant’s birth among participants who prepared the LHC (93% for father’s age and 94% for mother’s age) compared to those who did not (89% for both father’s and mother’s age). In contrast, data agreement for number of older siblings was comparable in both groups (96% among those who prepared the LHC and 97% among those who did not).

## Discussion

We found the preparation of a LHC and its consultation to be associated with higher data completeness despite low proportions of missing values in the overall questionnaire and enhanced data agreement between two distinct sources.

The discrepancy in data completeness between participants who prepared the LHC and those who did not was most noticeable in the *Date variables*. The LHC was designed as a visual representation of a timeline of events, with the expectation that it would help in recalling the exact timing of events. Although the number of missing values decreased with increasing consultation frequency of the LHC, the difference was more pronounced when comparing whether the LHC was prepared or not. It could be hypothesized that participants who have a better recollection of the information did not need to consult the LHC. Moreover, the preparation of a LHC may have primed the participants to think about their past, making them more inclined to answer every question from the main questionnaire.

The difference in data completeness between different data collection methods was partly due to our design. Those participating over the phone had more missing data. In fact, online participants were encouraged to provide answers due to the questionnaire web design, hence the low proportions of missing values. Indeed, a “Don’t know” answer option was not offered from the outset, but participants could still skip to the next question. In earlier studies using the LHC, participants were guided by trained interviewers. In our study, the phone interviewers were not aware of the content of the LHC; they were only instructed to remind participants to consult it as required. Interviewers from the Institut de la statistique du Québec were trained to follow a script, so they avoided subjectively influencing the participants’ answers.

Participants self-selected to complete the LHC. Those who chose to prepare it had a distinct profile compared to those who did not. To compare similar groups in our analyses, we used propensity scores and inverse probability of “treatment” weighting. Instead of simply adjusting for variables in the model, we used these weights in regression analyses. This approach tends to yield more cautious results. An overwhelming proportion of participants who prepared the LHC completed the questionnaire online, compared with three quarters of those who didn’t prepare it. This suggested that participants who prepared the LHC may have been more motivated to participate than the others, directly going to the online questionnaire. Among phone participants were individuals who were contacted by our interviewers, because they had not yet participated online. Consequently, we incorporated the mode of data collection as a confounding factor in our regression models. Despite our approach to adjust for these factors, we cannot exclude the presence of residual confounding or conclude about causality. The only approach that would allow to fully address these issues would be to randomly allocate participants to LHC completion or not. Since this methodological aspect was not the main goal of our study, no such approach was implemented.

Methodological studies on LHCs have primarily focused on aspects related to data reliability and accuracy, where all the participants completed the LHC [13, 15, 20, 21, 33, 34]. Previous studies highlighted that, in contrast to a conventional questionnaire to collect data, a LHC increased data accuracy. This was achieved by comparing the agreement between data collected with the LHC and public records [16, 20, 33, 34]. In our study, LHC preparation was associated with a slightly higher data agreement between participants’ reports and database records for their parents’ age, but not for the number of older siblings. The agreement for the latter was nearly perfect. The LHC was part of the recruitment documents which also included a list of difficult-to-remember information like parents’ age, breastfeeding or antibiotic use. The participants who completed the LHC may have been primed for the main questionnaire by this list. There were few variables available in both sources and these variables were not optimal, limiting the informativeness of these results.

Several studies have delved into the aspect of data completeness by quantifying the number of reported events, a higher count indicating greater completeness. In studies by Glasner et al. and by Morselli and Berchtold, where participants were randomized into either completing a conventional questionnaire or a LHC, no difference in the number of reported events was observed between groups [19, 20]. However, in Glasner et al.’s study, participants in the LHC group reported more periods of unemployment than in the conventional questionnaire group. This discrepancy could be attributed to the fact that unemployment is more challenging to recollect compared to events like employment or residential moves. In a more recent study in rural Nepal by Axinn et al., completion of a LHC before a questionnaire was related to a higher reported incidence of potentially traumatic events [11]. The LHC design was different in Glasner et al., Morselli and Berchtold, and Axinn et al.’s studies: in the former two, the LHC was self-administered online and replaced the conventional questionnaire; in the latter, a paper-version of the LHC was administered by a trained interviewer as a memory aid to the main questionnaire. Our results were consistent with the Nepalese study, showing more reported information (fewer missing values) among participants who completed a LHC [11]. The information in the LHC may be easier to remember, but it can serve as memory cues for domains covered in the main questionnaire that may be more difficult to recall. In Glasner’s study, the researchers also noted that the LHC aided in recalling more distant events but had less impact on more recent events [19]. We did not observe a difference in data completeness between more distant events (*Childhood variables*) and data as a whole (*Main variables*) when the LHC was utilized. The difference in findings may be explained by the nature of collected information, unemployment dates in their study and childhood events, more distant information, in ours.

Some limitations must be acknowledged. Factors associated with data completeness, such as individual personality traits (e.g., memory skills, attention to details, life organization) were not available, therefore were not considered. Additionally, certain socio-economic position variables employed in our study were contextually defined and temporally preceded data collection. The income and deprivation indices were based on the postal code of the participants’ residence in 2011 and 2014, whereas data collection occurred in 2021. We assumed that their socioeconomic situations did not undergo significant changes.

Our study’s strengths lie in the analytical approach, large sample size, and homogeneity of the population (particularly regarding ethnicity, age, and residential area type). The similar age of participants likely led to exposure to comparable life events. Their shared ethnic backgrounds and type of residential area might have resulted in similar responses to historical cues and/or life circumstances in the LHC. To our knowledge, our study is the first to examine data completeness within a questionnaire while using a self-completed LHC as memory aid and is the first to explore the impact of its consultation frequency during data collection.

## Conclusions

This study offers evidence that the incorporation of a LHC into data collection is linked to enhanced data completeness for retrospective exposures. However, our ability to evaluate data accuracy was limited due to the suboptimal nature and small number of available variables for comparison.

The LHC supports memory recall by facilitating reconnections across multiple life domains and serves as a potent memory aid to a conventional questionnaire in epidemiological research. A LHC appears to be particularly well-suited for capturing information related to changes in residences, shifts in occupations, and significant personal events. It may also be used to study the temporality of specific behaviors such as physical activity [35]. In epidemiological studies where data on multiple risk factors and intricate details are typically collected, it would not be very practical to gather all this information through a LHC. Therefore, a LHC may not readily replace a conventional questionnaire, but rather be a complementary tool, used during data collection to enhance recall and reduce missingness.

Future research should consider randomized trials to investigate the impact of using a LHC on data quality. Assigning one group of participants to a self-completed LHC would help mitigate residual confounding and self-selection bias which may have influenced our study despite efforts to control for these factors.

## Supplementary Information

Additional file 1. Table S1 – Variables used for the missing value count: List of variables used to count the number of missing values for the three variables groups [i.e. 1) Main variables (*n*=130); 2) Date variables (*n*=30); 3) Childhood variables (*n*=45)].

Additional file 2. Table S2 – Goodness-of-fit statistics for the Poisson, zero-inflated Poisson, negative binomial and zero-inflated negative binomial models for the relationship between the preparation of a life history calendar and the number of missing values in the data collection questionnaire for the 12 imputed datasets.

Additional file 3. Table S3 - Comparison of the negative binomial and a zero-inflated negative binomial models for the relationship between the preparation of a life history calendar and the number of missing values in the data collection questionnaire.

## Data Availability

Our work is governed by data privacy considerations, and the data are not publicly available. Some information and the computing code can be made available upon reasonable request.

## Abbreviations

CI: Confidence interval
LHC: Life history calendar
MICE: Multiple imputation by chained equation
SD: Standard deviation
SMD: Sample mean difference
SMR: Sample mean ratio
