# Supplementary material for: Use of a self-completed life history calendar in relation to data completeness and accuracy
Source: BMC Med Res Methodol. 2026 Feb 5;26:36. doi: 10.1186/s12874-026-02777-5 (PMC12896007; doi:10.1186/s12874-026-02777-5)
Supplement: Supplementary file 1 — Additional file 1. Table S1 – Variables used for the missing value count: List of variables used to count the number of missing values for the three variables groups [i.e. 1) Main variables (n=130); 2) Date variables (n=30); 3) Childhood variables (n=45)]. [file 12874_2026_2777_MOESM1_ESM.docx]

# Additional file 1

| **Table S1 – Variables used for the missing value counts: List of variables used to count the number of missing values for the three variables groups [*Main variables* (n=130); *Date variables* (n=30); *Childhood variables* (n=45)].** | | | |
| --- | --- | --- | --- |
| **Variables** | ***Main variables*** | ***Date variables*** | ***Childhood variables*** |
| Residence of the parents at birth of participant | X |  | X |
| Father’s occupation at birth of participant | X |  | X |
| Father’s occupation during participant’s childhood | X |  | X |
| Father’s occupation during participant’s adolescence | X |  |  |
| Mother’s occupation at birth of participant | X |  | X |
| Mother’s occupation during participant’s childhood | X |  | X |
| Mother’s occupation during participant’s adolescence | X |  |  |
| Childbirth type | X |  | X |
| Planned cesarean section | X |  | X |
| Breastfeeding (ever/never) | X |  | X |
| Duration of breastfeeding | X |  | X |
| Consumption of baby formula (ever/never) | X |  | X |
| Age first introduced solid food | X |  | X |
| Kindergarten or pre-school attendance | X |  | X |
| Start year of kindergarten (period 1) | X | X | X |
| End year of kindergarten (period 1) | X | X | X |
| Number of days at kindergarten (period 1) | X |  | X |
| Start year of kindergarten (period 2) | X | X | X |
| End year of kindergarten (period 2) | X | X | X |
| Number of days at kindergarten (period 2) | X |  | X |
| Ever had a pet | X |  |  |
| Year start of possession of pet 1 | X | X |  |
| Year end of possession of pet 1 | X | X |  |
| Year start of possession of pet 2 | X | X |  |
| Year end of possession of pet 2 | X | X |  |
| Ever smoked | X |  |  |
| Start year of 1st period cigarette smoking | X | X |  |
| End year of 1st period cigarette smoking | X | X |  |
| Quantity of cigarettes smoked during 1st period | X |  |  |
| Unit for quantity of cigarettes smoked during 1st period | X |  |  |
| Start year for another smoking period 1 | X | X |  |
| End year for another smoking period 1 | X | X |  |
| Quantity of cigarettes smoked during for another smoking period 1 | X |  |  |
| Unit for quantity of cigarettes smoked during for another smoking period 1 | X |  |  |
| Start year for another smoking period 2 | X | X |  |
| End year for another smoking period 2 | X | X |  |
| Quantity of cigarettes smoked during for another smoking period 2 | X |  |  |
| Unit for quantity of cigarettes smoked during for another smoking period 2 | X |  |  |
| Father smoked during pregnancy with participant | X |  |  |
| Mother smoked during pregnancy with participant | X |  |  |
| Smoking by surrounding people at home between age 0 and 3 years | X |  | X |
| Smoking by surrounding people at home between age 4 and 11 years | X |  | X |
| Smoking by surrounding people at home between age 12 and 18 years | X |  |  |
| Smoking by surrounding people at home between age 19 and 30 years | X |  |  |
| Smoking by surrounding people at home between age 31 years and in 2014 | X |  |  |
| Smoking by surrounding people at school or work between age 19 and 30 years | X |  |  |
| Smoking by surrounding people at school or work between age 31 years and in 2014 | X |  |  |
| Food consumption at 10 years old for each of the following foods: fruits, salad and raw vegetables, potatoes, other vegetables, milk, cheese and yogurt, refined grains, whole grains, poultry, cold cuts, beef/pork/other meats, seafood, legumes, eggs, nuts, pastries, chocolate and candies, soft drinks, fast food, salty snacks | X |  | X |
| Food consumption at 20 years old for each of the following foods: fruits, salad and raw vegetables, potatoes, other vegetables, milk, cheese and yogurt, refined grains, whole grains, poultry, cold cuts, beef/pork/other meats, seafood, legumes, eggs, nuts, pastries, chocolate and candies, soft drinks, fast food, salty snacks, tea, coffee, alcohol | X |  |  |
| Age started occasional alcohol drinking | X | X |  |
| Age started regular alcohol drinking | X | X |  |
| Physical activity level (leisure) between age 4 and 11 years | X |  | X |
| Physical activity level (leisure) between age 12 and 18 years | X |  |  |
| Physical activity level (leisure) between age 19 and 30 years | X |  |  |
| Physical activity level (leisure) between age 31 and age in 2014 | X |  |  |
| Physical activity level (occupational) between age 19 and 30 years | X |  |  |
| Physical activity level (occupational) between age 31 and age in 2014 | X |  |  |
| Ever had an illness of the digestive tube | X |  |  |
| Year of diagnosis digestive tube illness: celiac disease | X | X |  |
| Year of diagnosis digestive tube illness: H. pylori infection | X | X |  |
| Year of diagnosis digestive tube illness: irritable bowel syndrome | X | X |  |
| Year of diagnosis digestive tube illness: other | X | X |  |
| Surgical operation of the digestive tube (ever/never) | X |  |  |
| Year of surgery: appendicectomy | X | X |  |
| Year of surgery: bariatric | X | X |  |
| Year of surgery: colostomy | X | X |  |
| Year of surgery: intestine resection | X | X |  |
| Year of surgery: tonsillectomy | X | X |  |
| Year of surgery: other | X | X |  |
| Year of puberty for men | X | X |  |
| Year of puberty for women | X | X |  |
| Ever received an antibiotic treatment | X |  |  |
| Ever received an antibiotic treatment between age 0 and 3 years | X |  | X |
| Ever received an antibiotic treatment between age 4 and 11 years | X |  | X |
| Ever received an antibiotic treatment between age entre 12 and 18 years | X |  |  |
| Ever received an antibiotic treatment between age 19 and 30 years | X |  |  |
| Ever received an antibiotic treatment between age 31 and age in 2014 | X |  |  |
| Number of antibiotic treatments between age 0 and 3 years | X |  | X |
| Number of antibiotic treatments between age 4 and 11 years | X |  | X |
| Number of antibiotic treatments between age 12 and 18 years | X |  |  |
| Number of antibiotic treatments between age 19 and 30 years | X |  |  |
| Number of antibiotic treatments between age 31 and age in 2014 | X |  |  |
| Start year for the longest job | X | X |  |
| End year for the longest job | X | X |  |
