# Supplementary material for: Use of a self-completed life history calendar in relation to data completeness and accuracy
Source: BMC Med Res Methodol. 2026 Feb 5;26:36. doi: 10.1186/s12874-026-02777-5 (PMC12896007; doi:10.1186/s12874-026-02777-5)
Supplement: Supplementary file 2 — Additional file 2. Table S2 – Goodness-of-fit statistics for the Poisson, zero-inflated Poisson, negative binomial and zero-inflated negative binomial models for the relationship between the preparation of a life history calendar and the number of missing values in the data collection questionnaire for the 12 imputed datasets. [file 12874_2026_2777_MOESM2_ESM.docx]

# Additional file 2

| Table S2 – Goodness-of-fit statistics for the Poisson, zero-inflated Poisson, negative binomial and zero-inflated negative binomial models for the relationship between the preparation of a life history calendar and the number of missing values in the data collection questionnaire for the 12 imputed datasets | | | | | |
| --- | --- | --- | --- | --- | --- |
| Goodness-of-fit statistic | Dataset | Poisson regression | Zero-inflated Poisson regression^1^ | Negative binomial regression | Zero-inflated negative binomial regression^1^ |
| BIC | Dataset 1 | 8782.09 | 7299.29 | 5702.98 | 5712.88 |
|  | Dataset 2 | 8454.99 | 7102.09 | 5659.26 | 5668.35 |
|  | Dataset 3 | 8452.44 | 7099.17 | 5654.23 | 5660.70 |
|  | Dataset 4 | 8458.21 | 7107.28 | 5653.67 | 5661.05 |
|  | Dataset 5 | 8435.55 | 7098.01 | 5654.62 | 5663.56 |
|  | Dataset 6 | 9026.15 | 7447.92 | 5731.10 | 5741.97 |
|  | Dataset 7 | 8672.10 | 7231.24 | 5691.66 | 5697.56 |
|  | Dataset 8 | 8484.52 | 7120.33 | 5663.69 | 5672.48 |
|  | Dataset 9 | 8688.12 | 7237.55 | 5696.92 | 5705.97 |
|  | Dataset 10 | 8653.37 | 7224.27 | 5683.57 | 5691.28 |
|  | Dataset 11 | 8490.92 | 7117.48 | 5671.54 | 5679.48 |
|  | Dataset 12 | 8539.03 | 7150.55 | 5669.15 | 5678.69 |
| Compilation based on data from the ©Government of Québec, Institut de la statistique du Québec, CO·MMUNITY, 2017. ©Government of Québec, Institut de la statistique du Québec, Life History Intestinal Health Study, 2021. Institut de la statistique du Québec is not responsible for compilations or interpretation of results.  ^1^ Count part of the model: life history preparation (prepared, non-prepared) and mode of data collection (online, telephone). Zero-inflation part of the model: IBD status (Crohn’s disease, ulcerative colitis, controls), education (primary and secondary education, college, university) and residence area (urban, rural). These three latter variables were chosen because they presented a discrepancy in the proportion of zeros (for number of missing values) across the different groups. | | | | | |
