# Supplementary material for: Use of a self-completed life history calendar in relation to data completeness and accuracy
Source: BMC Med Res Methodol. 2026 Feb 5;26:36. doi: 10.1186/s12874-026-02777-5 (PMC12896007; doi:10.1186/s12874-026-02777-5)
Supplement: Supplementary file 3 — Additional file 3. Table S3 - Comparison of the negative binomial and a zero-inflated negative binomial models for the relationship between the preparation of a life history calendar and the number of missing values in the data collection questionnaire. [file 12874_2026_2777_MOESM3_ESM.docx]

# Additional file 3

| Table S3 - Comparison of the negative binomial and zero-inflated negative binomial models for the relationship between the preparation of a life history calendar and the number of missing values in the data collection questionnaire | |
| --- | --- |
| Model | Estimate result^1^ |
|  | Prepared Life history calendar (Reference: not prepared) |
| Negative binomial | SMR = 0.78 (95%CI= 0.64 - 0.96) |
| Zero-inflated negative binomial^2^ | IRR = 0.78 (95%CI = 0.64 – 0.95) |
| Compilation based on data from the ©Government of Québec, Institut de la statistique du Québec, CO·MMUNITY, 2017. ©Government of Québec, Institut de la statistique du Québec, Life History Intestinal Health Study, 2021. Institut de la statistique du Québec is not responsible for compilations or interpretation of results.  ^1^ Pooled results from the analysis of the 12 imputed datasets  ^2^ Count part of the model: life history preparation (prepared, non-prepared) and mode of data collection (online, telephone). Zero-inflation part of the model: IBD status (Crohn’s disease, ulcerative colitis, controls), education (primary and secondary education, college, university) and residence area (urban, rural). These three latter variables were chosen because they presented a discrepancy in the proportion of zeros (for number of missing values) across the different groups. | |
